# Supplementary material for: Top-down modulation of olfactory-guided behaviours by the anterior olfactory nucleus pars medialis and ventral hippocampus
Source: Nat Commun. 2016 Dec 22;7:13721. doi: 10.1038/ncomms13721 (PMC5192165; doi:10.1038/ncomms13721)
Supplement: Supplementary Information — Supplementary Figures 1-11. [file ncomms13721-s1.pdf]

Supplementary Figures

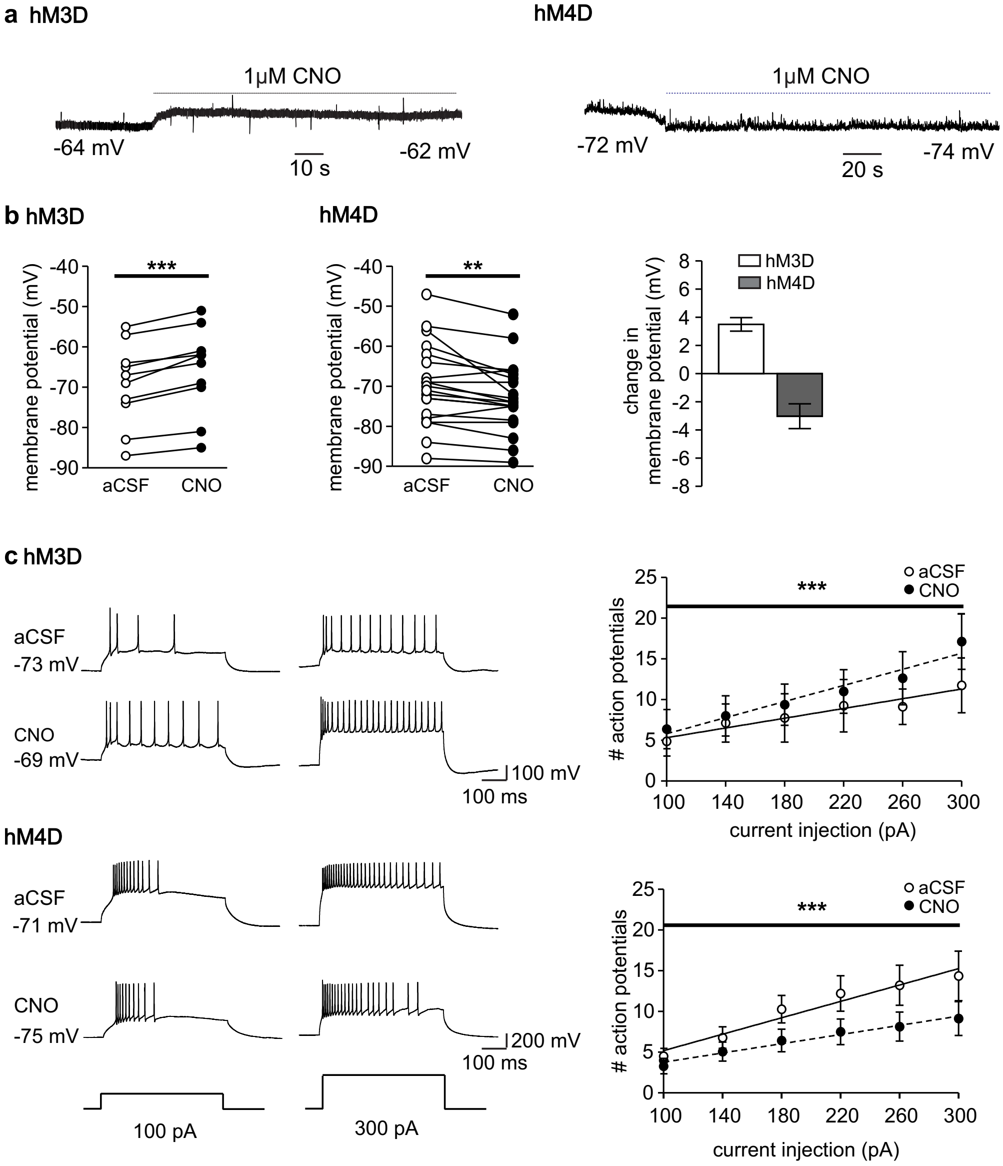

Supplementary Figure 1. Chemogenetic activation and inhibition of mAON neurons. **(a)** Representative traces from whole-cell patch clamp recordings taken from Left: hM3D-positive neuron and Right: hM4D-positive neuron in response to bath application of CNO (1 μM). **(b)** The effect of CNO on membrane potential in individual Left: hM3D-positive neurons (n=10, paired-samples t-test, ***P <0.001) and Middle: hM4D-positive neurons (n=20, paired-samples t-test, **P < 0.01). Right: CNO-induced change in membrane potential from baseline recorded in hM3D- and hM4D-positive neurons. CNO depolarized hM3D-expressing neurons and hyperpolarized hM4D-expressing neurons. **(c)** Left: Representative traces of evoked action potentials taken from Top: hM3D- and Bottom: hM4D-positive neurons under the 100 pA and 300 pA stimulus. Right: Relationship between the number of evoked action potentials and current injection (from 100 pA to 300 pA) in Top: hM3D- and Bottom: hM4D-positive neurons. CNO increased evoked action potential firing in hM3D-positive neurons (n=8, R^2=0.9313 for aCSF, R^2=0.9409 for CNO, two-away ANOVA, ***P<0.001) and suppressed evoked action potential firing in hM4D-positive neurons (n=14, R^2=0.9521 for aCSF, R^2=0.9723 for CNO, two-away ANOVA, ***P<0.001).

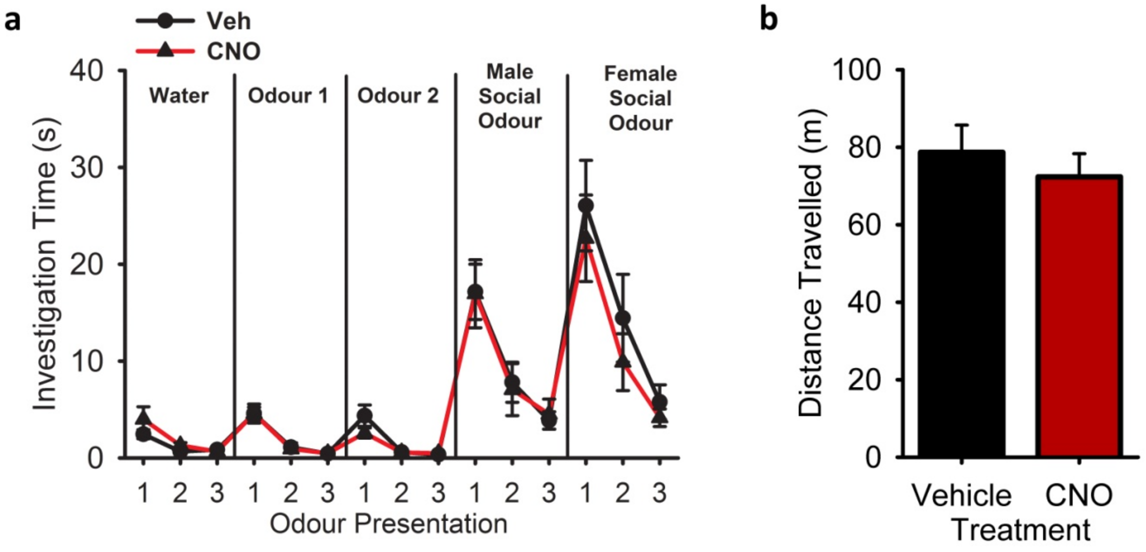

Supplementary Figure 2. hM4D-mediated inhibition of mAON activity has no effect on the novelty response to odours or locomotor activity. **(a)** CNO did not alter habituation to novel odours (n=10, two-way ANOVAs, all interactions $F_{(2,18)}<2.70$, ns, $P>0.095$; all main effects of trial $F_{(2,18)}>10.26$, $P<0.01$) or the reinstatement of novel odour investigation (all interactions $F_{(1,9)}<2.81$, ns, all main effects of trial $F_{(1,9)}>26.68$, $P<0.01$). **(b)** Open field locomotor activity was unaltered following CNO treatment
(n=10, paired-samples t-test, t(9)=1.67, ns, P=0.13).

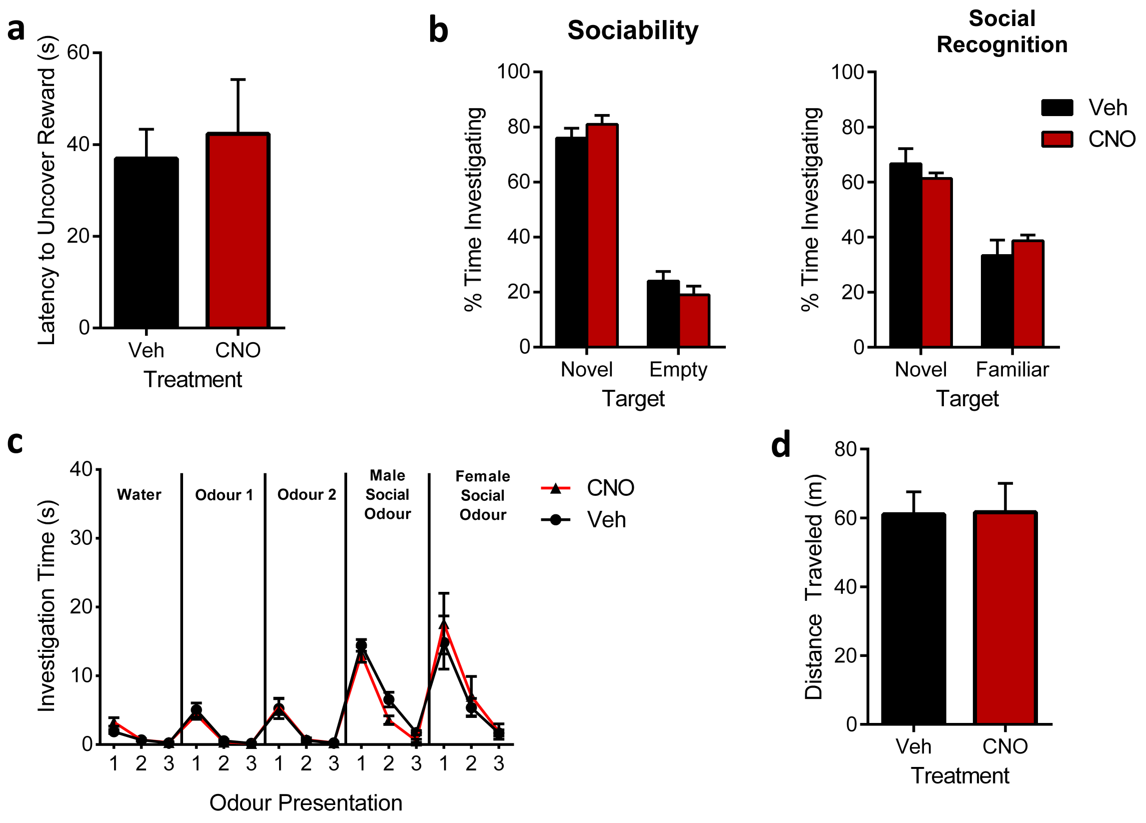

Supplementary Figure 3. Systemic injection of CNO has no effect on olfactory-dependent behaviours and locomotor activity in a sham surgery control group. For sham surgery control, a separate group of mice were prepared that underwent the same surgical procedures as mice in the hM3D or hM4D conditions, but did not express the transgenes in the AON. **(a)** CNO treatment did not influence the latency to locate a buried food reward in sham surgery animals (n=9, paired-samples t-test, $t_{(8)}$=0.56, ns, P=0.59). **(b)** Left: CNO-treated mice displayed no change in sociability (vehicle group n=5, CNO group n=5, independent-samples t-test, $t_{(8)}$=1.049, ns, P=0.32). Right: CNO-treated mice were able to distinguish between a novel and familiar conspecific (independent-samples t-test, $t_{(8)}$=0.90, ns, P=0.39). **(c)** CNO did not alter habituation to novel odours or the reinstatement of novel odour investigation (n=9, two-way ANOVAs, all interactions F(14, 120)<3.40, ns, P=0.36; all main effects of trial F(14, 120)>12.88, P<0.001). **(d)** Open field locomotor activity was unaffected by CNO treatment (n=11, paired-samples t-test, $t_{(10)}$=0.1364, ns, P=0.89).

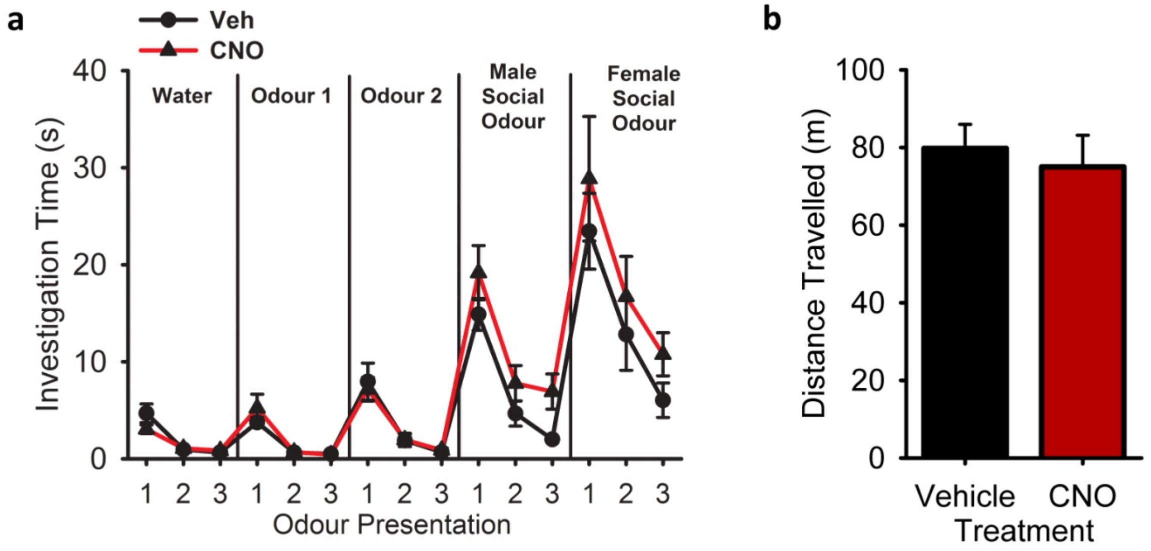

Supplementary Figure 4. hM3D-mediated activation of mAON activity has no effect on the novelty response to odours or locomotor activity. **(a)** CNO did not alter habituation to novel odours (n=10, two-way ANOVAs, all interactions $F_{(2,18)}<2.03$, ns, $P>0.16$; all main effects of trial $F_{(2,18)}>9.48$, $P<0.01$) or the reinstatement of novel odour investigation (all interactions $F_{(1,9)}<1.51$, ns, all main effects of trial $F_{(1,9)}>29.44$, $P<0.001$). **(b)** Open field locomotor activity was unaltered following CNO treatment (n=10, paired-samples t-test, $t_{(9)}=0.68$, ns, $P=0.51$).

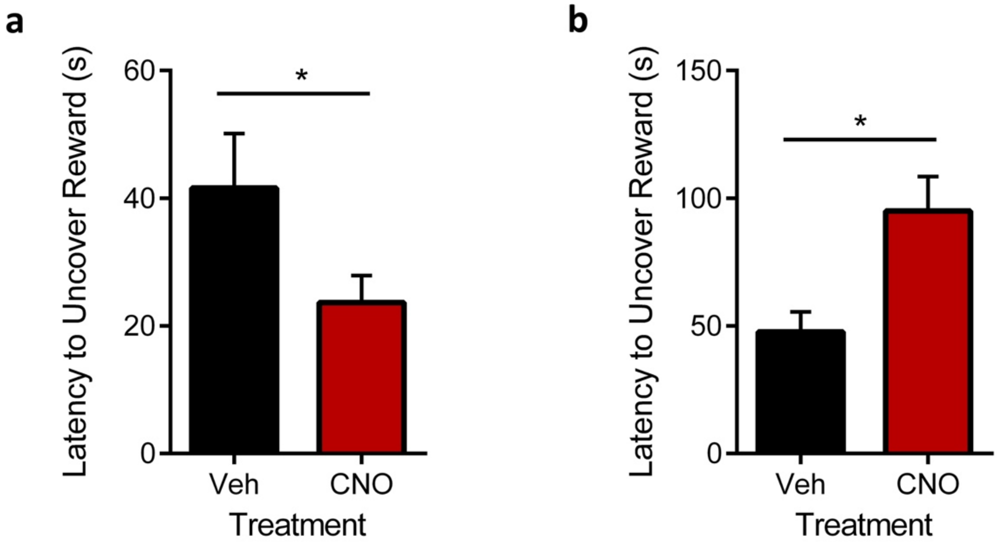

Supplementary Figure 5. A new group of hM4D or hM3D-expressing mice are capable of showing the effects of mAON manipulation on olfactory behavior. **(a)** For hM4D-expressing mice, CNO treatment decreased the latency locate a buried food reward (n=8, paired-samples t-test, $t_{(7)}$=2.52, *P<0.05) **(b)** For hM3D-expressing mice, CNO treatment increased the latency to locate a buried food reward (n=8, paired-samples t-test, $t_{(7)}$=2.70 *P<0.05).

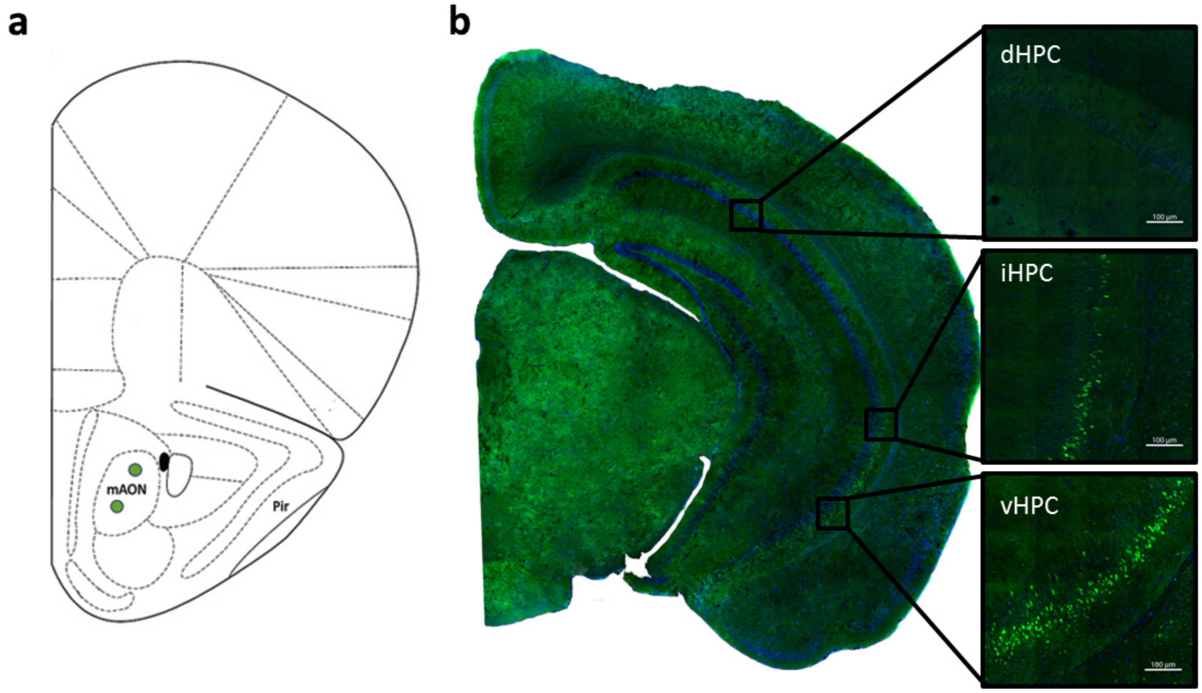

Supplementary Figure 6. A specific group of CA1 vHPC neurons projects to the mAON. **(a)** Green dots indicate infusion sites of the retrograde tracer Alexa488-conjugated CTB for two mice that underwent retrograde tracing experiments. **(b)** Transfer of CTB from mAON to the hippocampus of one representative mouse. Insets show confocal images of green CTB signal in cell bodies largely restricted to the CA1 of the vHPC, which ends abruptly at the rhinal fissure and is not found in the intermediate (iHPC) or dorsal CA1 (dHPC) of the hippocampus.

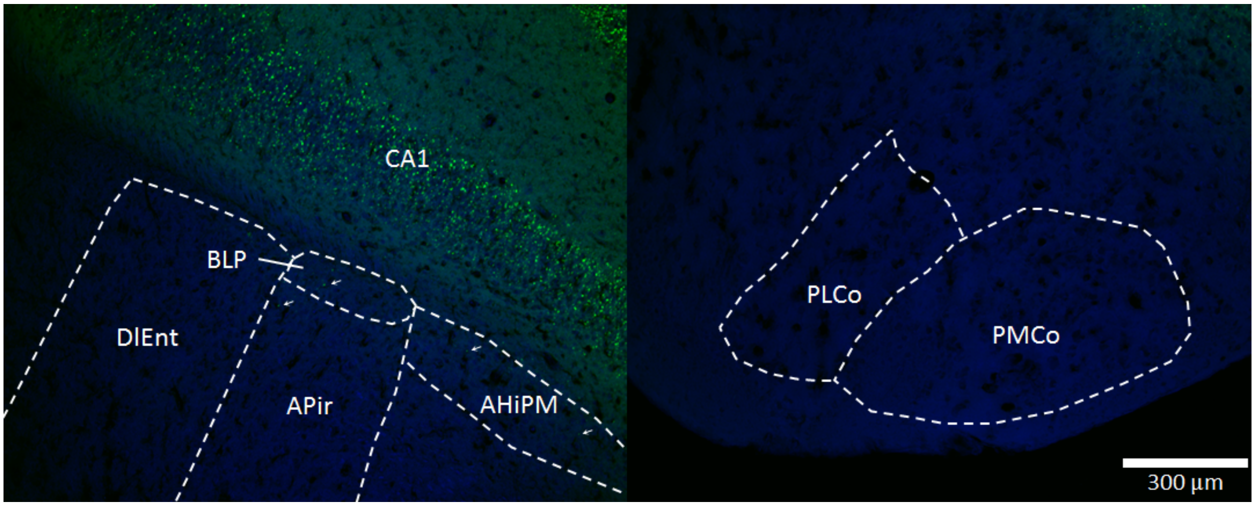

Supplementary Figure 7. AAV infection of cell bodies was mostly restricted to the vHPC. Any labeled cells outside of the vHPC were immediately lateral to the infected area of the vHPC (example cells are demarcated by white arrows; DIEnt, dorsointermedial entorhinal cortex, APir, amygdalopiriform transition, BLP, posterior part of the basolateral amygdala nucleus, AHiPM, posteromedial part of the amygdalohippocampal area). No cell bodies were observed in the poterolateral (PLCo) or posteromedial (PMCo) areas of the cortical amygdala.

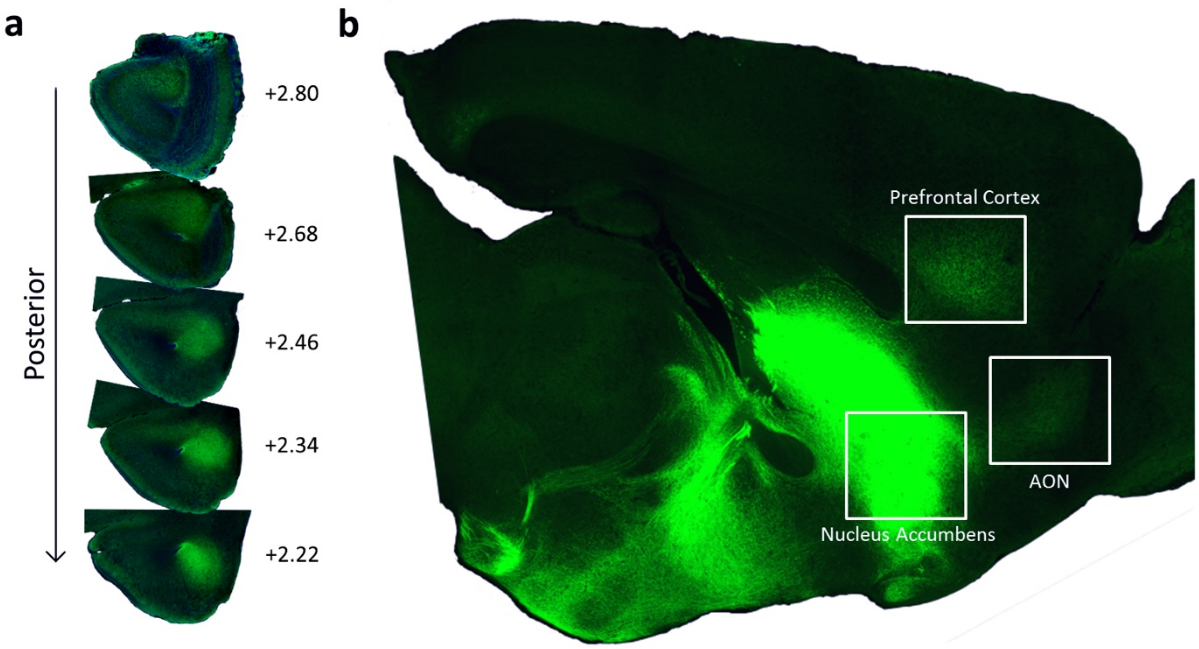

Supplementary Figure 8. Extent of vHPC terminal innervation of mAON visualized following AAVmediated expression of ChR2-YFP in the vHPC. **(a)** Coronal sections of the AON showing that vHPC terminals are most dense in posterior sections, and consistently restricted to the medial aspect of the AON. AP axis coordinates from bregma are presented right of the figure for reference. **(b)** A representative sagittal section showing vHPC terminal innervation of the forebrain.

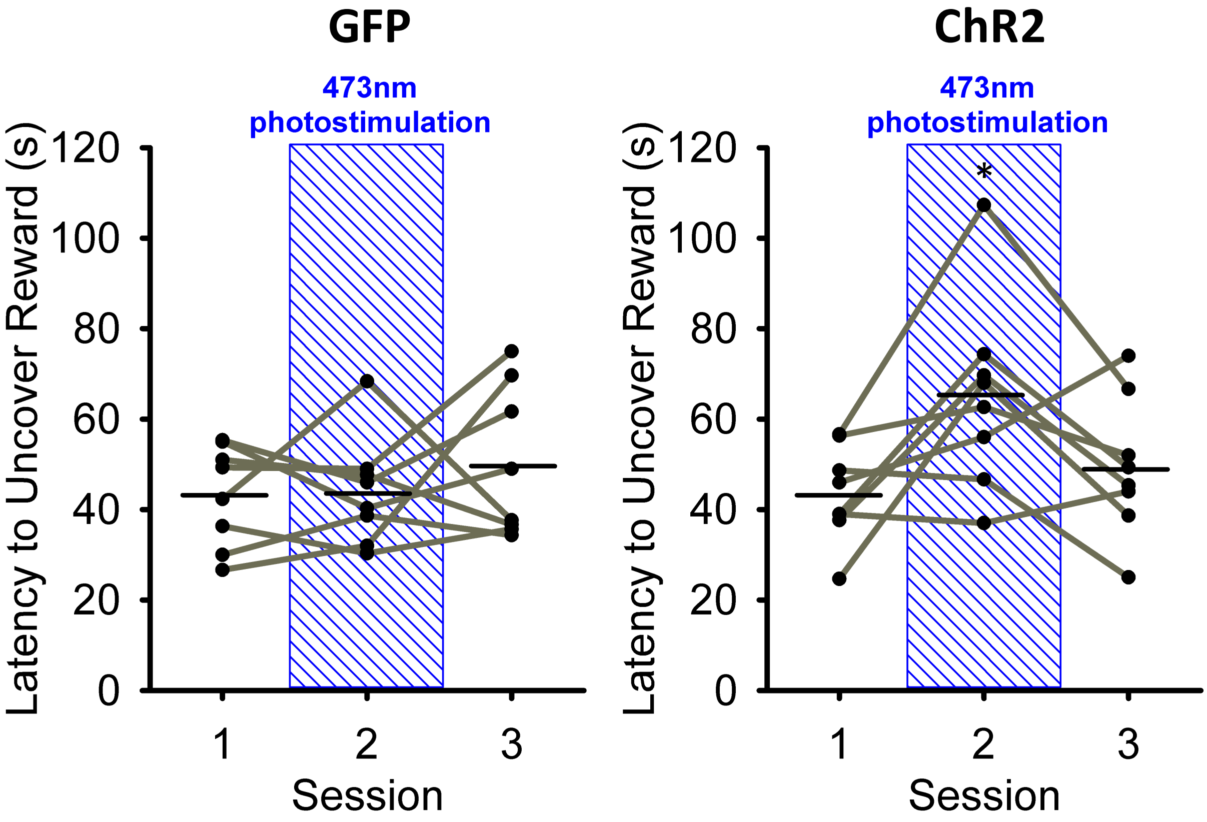

Supplementary Figure 9. Individual responses to vHPC-mAON pathway stimulation during performance of the buried food test. Black bars indicate mean latency. Stimulation significantly increased the latency to locate a buried food reward compared to control mice (two-way ANOVA interaction $F_{(2,28)}=3.60$, $P<0.05$; independent samples t-test session 2, $t_{(14)}=2.47$, $P<0.05$).

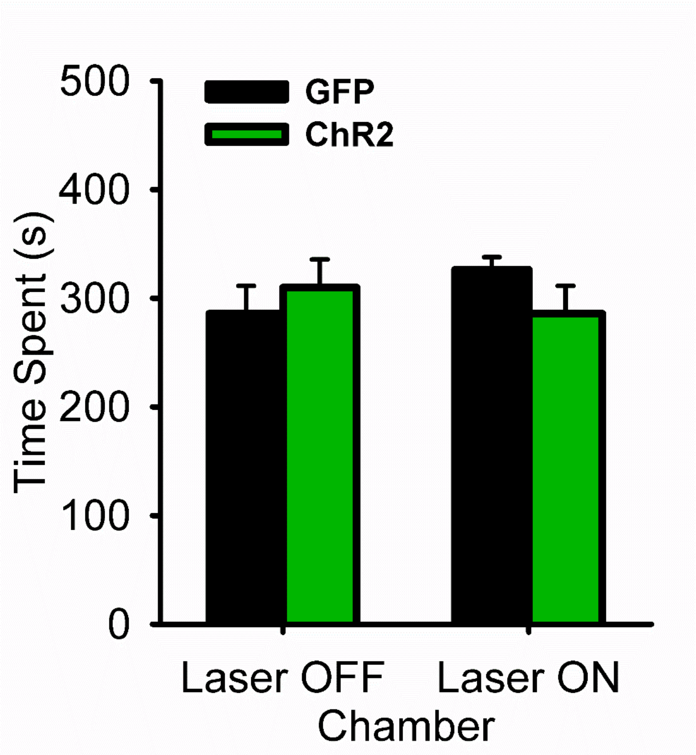

Supplementary Figure 10. Photostimulation of ChR2-containing vHPC terminals in the mAON does not serve as an appetitive or aversive stimulus. ChR2 mice spent equal amounts of time in a chamber paired with laser stimulation as a chamber without stimulation (n=8 per group, two-way ANOVA interaction $F_{(1,13)}$=2.3, ns, P=0.15).

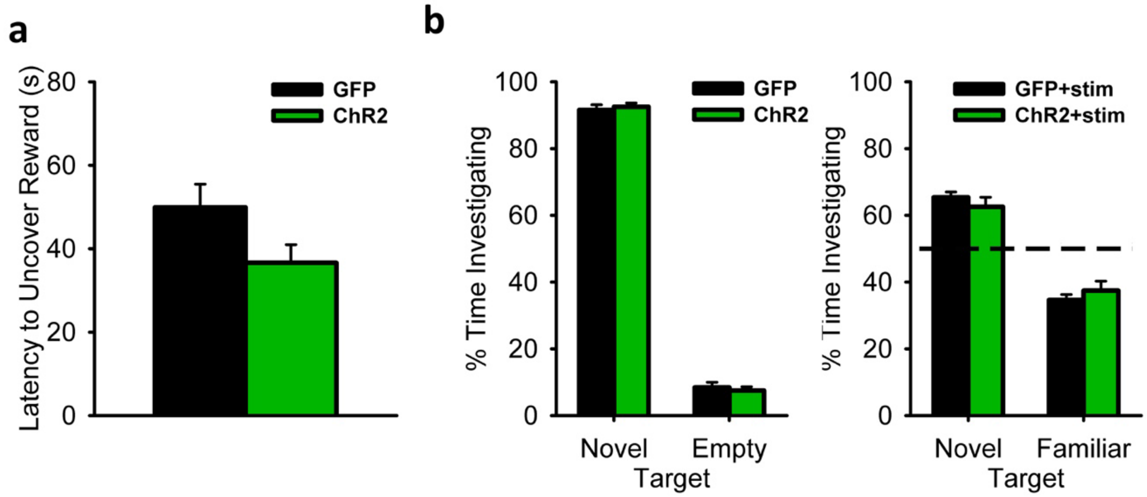

Supplementary Figure 11. (a) Photostimulation of ChR2-containing vHPC terminals in the PFC had no effect on the latency to uncover a buried food reward (GFP n=6, ChR2 n=8; independentsamples t-test, $t_{(12)}$=1.91, ns, P=0.08). **(b)** Left: In the absence of photostimulation, ChR2 and GFP mice spent a similar proportion of time investigating a novel conspecific vs. an empty cage (both groups n=8; independent-samples t-test, $t_{(14)}$=0.49, ns, P=0.62). Right: Photostimulation of vHPC inputs to the PFC had no effect on the ability of ChR2 mice to distinguish a novel and familiar conspecific ($t_{(14)}$=0.85, ns, P=0.41).
